# Transcriptome characterization of the South African abalone *Haliotis midae *using sequencing-by-synthesis

**DOI:** 10.1186/1756-0500-4-59

**Published:** 2011-03-11

**Authors:** Paolo Franchini, Mathilde van der Merwe, Rouvay Roodt-Wilding

**Affiliations:** 1 Molecular Aquatic Research Group, Department of Genetics, Stellenbosch University, Private Bag X1, Matieland, 7602, South Africa

## Abstract

**Background:**

Worldwide, the genus *Haliotis *is represented by 56 extant species and several of these are commercially cultured. Among the six abalone species found in South Africa, *Haliotis midae *is the only aquacultured species. Despite its economic importance, genomic sequence resources for *H. midae*, and for abalone in general, are still scarce. Next generation sequencing technologies provide a fast and efficient tool to generate large sequence collections that can be used to characterize the transcriptome and identify expressed genes associated with economically important traits like growth and disease resistance.

**Results:**

More than 25 million short reads generated by the Illumina Genome Analyzer were *de novo *assembled in 22,761 contigs with an average size of 260 bp. With a stringent *E*-value threshold of 10^-10^, 3,841 contigs (16.8%) had a BLAST homologous match against the Genbank non-redundant (NR) protein database. Most of these sequences were annotated using the gene ontology (GO) and eukaryotic orthologous groups of proteins (KOG) databases and assigned to various functional categories. According to annotation results, many gene families involved in immune response were identified. Thousands of simple sequence repeats (SSR) and single nucleotide polymorphisms (SNP) were detected. Setting stringent parameters to ensure a high probability of amplification, 420 primer pairs in 181 contigs containing SSR loci were designed.

**Conclusion:**

This data represents the most comprehensive genomic resource for the South African abalone *H. midae *to date. The amount of assembled sequences demonstrated the utility of the Illumina sequencing technology in the transcriptome characterization of a non-model species. It allowed the development of several markers and the identification of promising candidate genes for future studies on population and functional genomics in *H. midae *and in other abalone species.

## Background

Abalones (*Haliotis *spp., Haliotidae) are important fishery resources worldwide, with high commercial value. Because of their sedentary lifestyle along shallow rocky coastlines [1], these gastropods are vulnerable to capture. The subsequent over-exploitation resulted in a substantial decrease of wild populations. To compensate for the high demand of abalone, farming by means of aquaculture has been developed for several *Haliotis *species in different regions of their distribution area. Currently, abalone aquaculture is practiced on 12 species in 16 different countries [2].

*Haliotis midae *is one of the six endemic abalone species of southern Africa. Being the largest and most abundant South African species, this abalone is the ideal candidate for aquaculture and it is the only one to be commercially cultivated in the region. Commercial production in South Africa started in the 1980 s with annual production of farmed animals reaching 934 tons by 2008 [3].

In recent years, several genetic management procedures have been developed to optimize the production of various aquaculture species, mainly represented by fish and shellfish [4-7] and numerous molecular marker systems have been developed to assist these projects [8]. Notably, extensive research has been done in the field of marker assisted selection (MAS), with the aim to identify genetic markers surrounding quantitative trait loci (QTL). Quantitative variation, controlled by QTL and environmental influences, characterizes economically important traits in farmed animals, such as growth, meat quality and disease resistance. In aquaculture, this information can be utilized to maximise the rate of genetic gain from selective breeding programs [6,9].

Given the growing importance of abalone in the South African aquaculture market, a genetic improvement program was initiated in 2006 funded by government and industry. Since its inception, this program has aimed to increase productivity by exploiting the inherent biological potential of cultured populations using reproductive, quantitative and molecular approaches [2,10-14].

Despite its importance in aquaculture, genomic sequence resources for *H. midae*, and for abalones in general, are still scarce; as is the case for most non-model organisms. Next-generation sequencing technologies offer novel and rapid ways for genome-wide characterization and profiling of mRNAs, small RNAs, transcription factor regions, structure of chromatin, DNA methylation patterns and metagenomics [15]. This technology provides an efficient way to generate sequence data for non-model organisms in the form of transcriptome sequencing. Even though the transcriptome, or Expressed Sequence Tags (ESTs), represents a subset of the entire genome of eukaryotes, its sequencing is a valid alternative to whole genome sequencing. Advantages of investigating the transcriptome rather than the genome of an organism include focusing on the part of the genome with high functional information content [16] and avoiding introns and intragenic regions that can complicate the analysis of data [17]. Among the few companies that provide next generation genome analyzers, the Illumina Solexa sequencing-by-synthesis system has been widely used in transcriptome sequencing of organisms whose genomes are available [18-21] and in non-model organisms with no reference genomic resources [22]. Furthermore, the short reads produced by the Illumina Genome Analyzer (25-30 bp with the first models, 75-100 bp with the latest model) enabled reliable *de novo *assemblies into longer contigs useful for gene discovery, digital gene expression profiles and comparative genomics studies [22-26].

In this study, the Illumina Genome Analyzer II technology was used to generate over 1.1 billion bases of high quality DNA to characterize, for the first time, the *H. midae *transcriptome. We demonstrated the suitability of short-read sequencing for *de novo *assembly that generated more than 20,000 contigs. A substantial percentage of these rendered BLAST matches to known annotated genes in public databases. Furthermore, we explored the assembled transcripts in order to evaluate the level and distribution of transcriptome coverage in the main functional gene categories of various databases. Several transcripts involved in disease resistance, an economically important trait in abalone, were extracted to assist future studies on selective breeding and genetic engineering. Finally, we used the *de novo *assembled contigs to detect molecular markers (SNP and SSR). For a subset of those SSRs that satisfied strict amplification parameters, we designed high quality PCR primers in order to immediately provide molecular markers for use in genetic studies of *H. midae *and to test in cross amplification with other phylogenetically related species.

## Results and Discussion

### Assembly

Three different *H. midae *libraries were subjected to a run in three different lanes of the Illumina Genome Analyzer II. In the first lane, a cDNA library of three adult animals was sequenced. In the second and third lanes, two cDNA libraries originating from two different groups of eight adult animals of the same age, but with significant differences in weight and size, were sequenced. The non-normalised libraries allowed the study of differentially expressed genes in relation to growth, but the use of the whole body tissue as source of RNA prevented the study of tissue specific gene expression profiles. Differential expression analysis was the subject of a parallel project and will not be elaborated upon in this paper.

**Figure 1:**
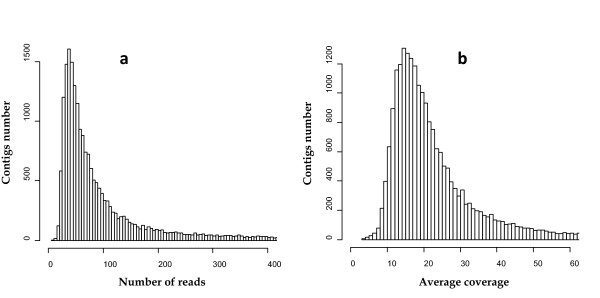
**Assembly statistics**. Histograms showing the distribution of the number of reads (a) and the average coverage of the assembled contigs (b).

After cleaning the data, the first lane's output yielded 2x(5,399,167) paired reads 40 and 45 bp long; the other two lanes yielded respectively 8,487,354 and 5,975,556 single end reads of 45 bp. A total of 25,261,244 reads were imported into the CLC Genomics Workbench for *de novo *assembly. Considering only contigs with a minimum length of 100 bp, the assembly rendered 21,761 sequences (utilizing 10,635,178 reads). Non-assembled reads and shorter contigs were discarded from further analyses since they could contain artefacts derived from cDNA synthesis, sequencing and contamination. The contigs ranged in size between the minimum set threshold of 100 bp and 10,744 bp (average size of 260 bp and a N50 value of 356 bp) with 2,394 contigs that were more than 500 bp in length. The number of reads per contig ranged between 9 and 143,200 with an average of 401 reads per contig (Figure 1a). The average nucleotide-wide coverage was estimated to 36.6x (Figure 1b). The contigs file is provided as Additional file 1 in *fasta *format.

The genome size of *H. midae *was recently described (C = 1.43 pg; 2803 MB), but genomic studies on this non-model species are in an early stage, and the percentage of its genome that is transcribed is unknown. This, together with the lack of genomic information in other abalone species (in which a substantial variation in genome size has also been observed: C-value ranges from 1.43 pg in *H. midae *to 2.14 pg in *H. corrugata*) make it difficult to predict how deep our *de novo *assembled sequences cover the South African abalone transcriptome [27].

Before evaluating the assembly by BLAST analysis against public protein databases, the quality was assessed by self-BLAST that rendered a low percentage of matches against other contigs with 100% identity and *E*-value <10^-50 ^(795 matches corresponding to 3.5% of total sequences), indicating the efficiency of the assembly of the Illumina short reads. Furthermore, in matches with 100% identity the alignment mostly extended for a short portion of the query (435 matches had alignment length ≤ 50 bp averaging 8% of the length of the query contig). These short alignments could indicate the presence of short repeated sequences, commonly spread across the eukaryotic genome, while the longer matches could indicate the occurrence of different transcripts of the same gene resulting from alternative splicing events [28,29].

### BLAST and Annotation

**Figure 2:**
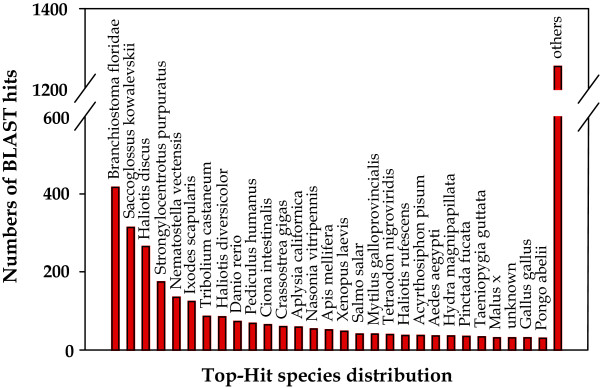
**Distribution of the BLAST matches**. Each bar of the histogram indicates the number of top-BLAST matches (the matches with the lowest *E*-value for each contig) against the Genbank non-redundant (NR) protein database to various species.

For the *de novo *assembly, where no annotated reference is available, the matching of contigs to known proteins gives an indication of the quality of assembly [30]. Using a stringent *E*-value threshold of 10^-10^, 3,841 out of 22,761 contigs (16.8%) had a BLAST homologous match against the NR protein database. This level of sequence similarity matching is low, but comparable to those found in other studies [22,31,32] where high throughput sequencing technology was used for the *de novo *transcriptome assembly of non-model species. The main reason for this result is probably the lack of large scale genomic resources for the genus *Haliotis *and other evolutionary related molluscs. In fact, even though several mollusc species are present in the top-BLAST match species distribution (the matches with the lowest *E*-value for each contig), other more distant taxa with comprehensive genomic resources in Genbank are present as well, both invertebrate and vertebrate (Figure 2).

**Figure 3:**
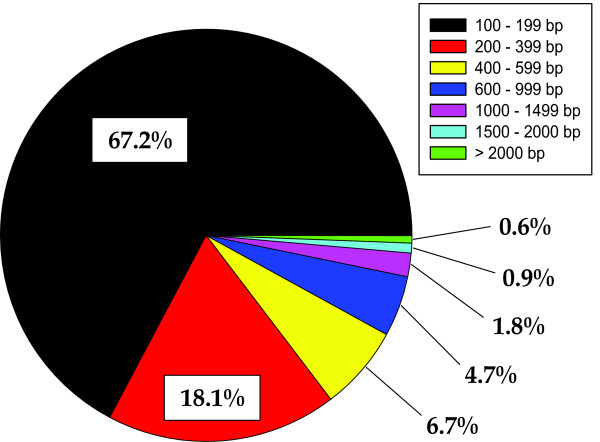
**Contigs length distribution**. The pie chart shows the percentage of contigs with the length range indicated in the upper legend.

The poor representation of abalones and closely related molluscs in public databases is also revealed by the BLAST analyses results when comparing *H. midae *with *Lottia gigantea*, a closely related mollusc with comprehensive genomic resources. The EST collection and the filtered genes of *Lottia *were used as reference during the sequence similarity search. The genera *Haliotis *and *Lottia *belong to different taxonomic groups (respectively Vetigastropoda and Patellogastropoda), and their evolutionary distance inferred by morphological and molecular data [33,34] is emphasized by the minimal percentage of gene similarity obtained by the BLAST analysis (15.2% to *Lottia *ESTs; 17.3% to *Lottia *predicted gene models). To confirm this pattern, mapping of *H. midae *short reads against the *Lottia *genome resources was only successful for a small subset of the reads (ranging approximately from 0.06% reads mapped to the *Lottia *ESTs and clustered ESTs to 0.07% reads mapped to the *Lottia *genes). Another explanation for the poor matching is represented by the abundance of short sequences in the contigs file. As shown in Figure 3, 15,285 sequences (67.2%) range between 100 and 200 bp and 19,414 (85.3%) are less than 400 bp in length. This is an expected result as, according to the BLAST algorithm, shorter sequences are required to have higher identity in order to satisfy a certain *E*-value.

The phylogenetic bias emerging from the BLAST analysis when using the NR database is not confirmed when the contigs were searched for similarity using BLASTn against the EST database collection of NCBI (dbEST), where a large number of EST sequences from non-model organisms is present (to date, only 70 ESTs of *H. midae *have been submitted in dbEST). Of the 8,190 sequences (36.0%) where similarity was found (*E*-value threshold of 10^-10^), 5,693 (69.5% of the matches) had top-BLAST matches against species of the genus *Haliotis *and 1,478 (18.0% of the matches) against other mollusc species. This finding is a further confirmation of the quality of our assembly.

The output of the BLAST analysis against the NR database is provided as Additional file 2 where 5,969 contigs matched known proteins (*E*-value threshold of 10^-3^). From this file, the above reported 3,841 matches were filtered according to a more stringent *E*-value threshold of 10^-10^. This stringent *E*-value allowed the annotation of these sequences to their described functions in public databases with a high level of confidence.

To classify the function of the predicted *H. midae *genes, GO annotation was performed. Out of the total 3,841 sequences with BLAST matches, Blast2GO successfully annotated 2,991 sequences associating them with 20,054 GO terms. Of these, 8,429 were assigned to the functional category 'Biological Process' (42.1%), 6,674 to 'Molecular Function' (33.2%) and 4,951 to 'Cellular Component' (24.7%). These contigs were assigned to most of the subcategories of the GO database.

**Figure 4:**
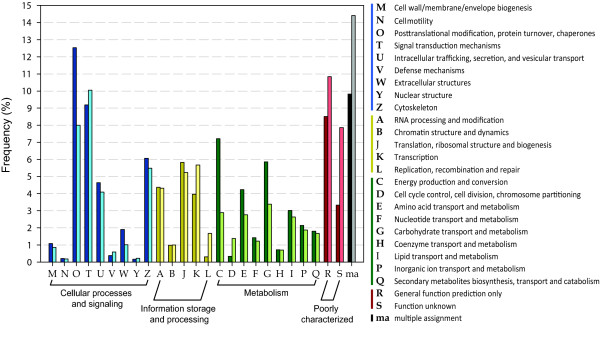
**Functional assignments to the KOG categories**. The graph shows the assignment of the *Haliotis midae *contigs and the *Lottia gigantea *clustered ESTs to the 25 categories of the eukaryotic orthologous groups of proteins (KOG). The main KOG categories are represented with different colours. Darker variants of each colour indicate the *H. midae *matches while lighter variants those of *L. gigantea*.

Functional annotation against the KOG database rendered 2,951 sequences that had BLAST matches with an *E*-value < 10^-10 ^(Figure 4). These were uniquely assigned to the four main KOG classes (ranging from 1,067 contigs, 36.2%, for the "Cellular Processes and Signaling" to 349 contigs, 11.8%, for "Poorly characterized") and each of their sub-categories were populated by at least five sequences. The main represented sub-categories were "Posttranslational modification, protein turnover, chaperones" (370 contigs), "Signal transduction mechanisms" (271), both belonging to the "Cellular Processes and Signaling" main class, and "Energy production and conversion" (213) of the "Metabolism" main class. Approximately 10% of the contigs (290) were assigned to more than one sub-category, and were thus indicated as "multiple assignment" in Figure 4. The distribution of the contigs in various functional classes of GO and KOG databases indicates how the transcriptome data, even though not covering the entire *H. midae *transcriptome, encompasses a broad gene diversity. To further evaluate this finding, the clustered ESTs of *L. gigantea *were searched for sequence similarity against the KOG database using BLASTx. Using the same *E*-value threshold of 10^-10^, this analysis rendered 10,056 annotated sequences with the assignment proportions to the KOG categories mirroring those of *H. midae *(Figure 4). The higher number of annotated ESTs for *L. gigantea *is an expected result because of the large amount of data sequenced; the most comprehensive among Gastropod taxa. Nevertheless, the similarity in KOG category distribution could reflect the adequate representation of the complete *H. midae *transcriptome.

### Disease resistance as relevant trait for aquaculture

Since the data generated potentially contained several genes associated with economically important traits such as disease resistance, the BLAST matches against the NR database, combined with the functional annotation to GO, KOG and PFAM databases were used to search for these specific transcripts. The selected trait can be harnessed in farmed abalone during selective breeding programs and in future, possibly through genetic engineering in order to optimize the production in terms of body mass and meat quality. Despite their fundamental role in aquaculture, only a few studies of quantitative traits in aquaculture species have been conducted (Abalone: [9]; Trout: [35]; Salmon: [36]). In recent years, the genomic revolution has allowed researchers to acknowledge the contribution of candidate genes [37] and many studies are now directed to increase the knowledge on the genetic base of these traits.

Infectious diseases are considered one of the main barriers to the successful development and continuation of molluscan aquaculture as they limit production in terms of quality, quantity and regularity [38,39]. Various investigations have revealed that infectious diseases can have a significant negative impact on the abalone aquaculture [40-47]. Different kinds of organisms are associated with diseases in cultured abalone. A protozoan of the group haplosporidian was associated with high mortalities of cultured juvenile paua (*Haliotis iris*) in New Zealand [46]. Various bacteria have also been isolated from cultured abalone experiencing disease and mortalities. Disease outbreaks among cultured abalone (*Haliotis rubra*, *H. laevigata *and their hybrids) in Tasmania, Australia, were associated with two species of *Vibrio *(*V. harveyi *and *V. splendidus *I) and a *Flavobacterium*-like bacterium [48]. In Japan, the bacterium *Vibrio carchariae *was isolated from cultured abalone (*Haliotis diversicolor supratexta*) experiencing a mass mortality [43]. Since 1998, *Vibrio harveyi*, a marine pathogen that affects a large range of animals, was responsible for episodic abalone epidemics associated with massive mortalities in France, Japan, and Australia, both in wild and farmed animals [49]. Currently, Tasmania is experiencing an outbreak of abalone Viral Ganglioneuritis (AVG), that after its first appearance in 2008, lead to the quarantining of land-based culture and processing facilities as well as closure of the local recreational fishery in January 2011 [50]. In *H. midae*, farmed animals experienced reduced growth rates due to sabellid polychaete infection [44].

Notwithstanding the relevant negative impact of various kinds of infections documented worldwide in wild and farmed animals, the biological pathways of stress response and disease resistance are not well characterized, and their genetic basis and control are still poorly understood.

Internal defense mechanisms against pathogens and parasites are generally constituted by cellular and soluble (humoral) haemolymph components. In terms of this latter immune system, different gene families related to various mechanisms involved in disease-resistance were found in the *H. midae *data spanning from those coding for lysosomal enzymes, lectins, and antimicrobial peptides (AMPs) to peptides mediating apoptosis and other genes regulating or acting on immune response. Several transcripts were annotated to genes coding for various kinds of lysosomal enzymes, such as acid or alkaline phosphatase, lipase, aminopeptidase and lysozyme enzymes that actively participate in parasite destruction (see Additional file 3). AMPs aid in recognition of pathogens and parasites by marking them for destruction via opsonising or direct killing [51]. Despite variations in structure and size, their role in anticancer activities, regulation of cell proliferation, wound healing effects and many cellular immune responses regulated by inducing gene expression was shown in several studies [52-54]. AMPs are well studied in molluscs and their possible use in aquaculture as antibiotics substitutes (resistance of bacteria to antibiotics is drastically increasing) is being evaluated [55]. One transcript was annotated to an AMP and two transcripts were found to be orthologous to H2A, a histone from which a potential antimicrobial peptide, Abhisin, is derived, as reported in a recent study on disk abalone, *Haliotis discus discus *[56]. Apoptosis is a highly conserved multifunctional process that not only plays a critical role in cellular and tissue homeostasis and embryonic development, but is also involved in the immune system where it limits possible damage caused by pathogens or parasites [57]. Many transcripts were found to code for regulators of apoptosis and they can be useful candidates to further elucidate this mechanism in *H. midae*.

Despite the importance of humoral defense mechanisms, cellular immunity seems to perform the main role in shellfish immune processes [58]. The main defence mechanism of molluscan haemocytes is the phagocytosis of pathogens; destroying them by the release of nitric oxide and lysosomal enzymes [59]. In both immune system mechanisms, host-pathogen recognition is a crucial step to activate the immune response. Several transcripts were homologous to genes coding for receptors involved in host-pathogen recognition (e.g. peptidoglycan recognition proteins, thrombospondin-like glycoproteins, scavenger receptors and contactin associated proteins) and to genes promoting the transduction of the signal (coding for peptides involved in, for example, kinase and notch signaling pathways).

Given the importance of this trait in cultured species such as the South African abalone *H. midae*, a brief list of transcripts expected to play a role in disease resistance was extracted from the data and is reported in Additional file 3 along with respective functional descriptions retrieved from the GO, KOG and PFAM databases. Although by no means exhaustive, this list of transcripts can represent a basis from where to identify candidate disease resistance genes for future studies on functional and comparative genomics. For such studies to be useful, however, a more thorough platform describing disease resistance pathways in molluscs is needed.

### Marker discovery

A total of 7,831 SSRs with a minimum of four contiguous repeat units (motifs range from two to six) were identified in 4,707 contigs (20.7%). The most abundant SSRs are characterized by dinucleotide motifs (6,059), followed by trinucleotide (1,103), tetranucleotide (547), hexanucleotide (67) and pentanucleotide (55) motifs (Table 1). To provide an immediate resource for future studies, primer pairs were designed in those contigs containing adequate flanking regions. After screening with more stringent search parameters (see Material and Methods), 420 primer pairs were found in 181 contigs (available in Additional file 4 in *fasta *format). Additional file 5 provides the information of each SSR including repeat motif, number of repeats, sequences of forward and reverse primer and melting temperature. Of the 4,707 contigs containing SSRs, 1,158 (24.6%) were found in annotated sequences. A lower percentage of annotated sequences were found for the contigs where primers were designed (8.8%, 16 out of 181). To validate the quality of this method for SSR discovery, a set of microsatellite loci selected from contigs assembled *de novo *from the reads of the first three animals (first lane of the sequencing) was tested for amplification [60]. Twenty two out of 27 tested primers gave a PCR product of the expected size and 14 of them were polymorphic. The SSR sequences where amplification was successful were also found in the final set of contigs obtained from the 19 animals, but they were not found in the 181 contigs where the 420 primers, reported in the Additional file 5 were designed. Since more stringent amplification conditions were applied to design these latter primers (in sequences with higher confidence), we expect a higher amplification efficiency from this final set.

**Table 1:** Summary statistics for the SSRs found in the *H. midae *library.

SSR type	SSR Number (4,707 contigs) Count/Percentage	SSR Number (181 contigs) Count/Percentage
Dinucleotide	6,059/77.4%	246/58.6%
Trinucleotide	1,103/14.1%	56/13.3%
Tetranucleotide	547/6.9%	96/22.9%
Pentanucleotide	55/0.7%	7/1.7%
Hexanucleotide	67/0.9%	15/3.5%

Total	7,831	420

The second column indicates all the SSRs with at least four motif repetitions and the third column, the loci where primer pairs with stringent criteria were designed (see Material and Methods section).

Currently, 215 microsatellite markers are available for *H. midae*, discovered either with traditional methods [13,14,61,62] or by pyrosequencing technology [2]. These are used for studies on population structure, genetic diversity, parentage, linkage mapping and QTL-mapping [2,63,64]. Thirty one of the available 215 microsatellites were found in our contigs. This apparently low match could be mainly explained by the fact that most of the available SSRs are found in genomic material of which only a sub-portion is transcribed. Furthermore, the complete transcriptome of *H. midae *could have been only partially covered by the *de novo *assembled contigs of this study and some of the EST-SSRs previously detected could be contained in sequences not covered by our assembly.

Traditional methods, for example the most widely used Fast Isolation by AFLP of Sequences Containing Repeats (FIASCO; [65]) and the SNX-unilinker method [66], are not suited for high-throughput development of markers because their use is expensive and time consuming. Next generation sequencing gives a superior resource for marker discovery mainly because of the large amount of data produced in a short time. Pyrosequencing of genomic DNA provided, for the first time, short sequence reads in *H. midae *and the microsatellite markers identified were used, together with other markers, to construct a preliminary linkage map for QTL detection [2]. The microsatellites identified in the present work have the advantage to be EST-based SSRs, therefore part of, or adjacent to functional genes. EST-SSRs are characterized by higher amplification rates and cross-species transferability in comparison to SSRs contained in non transcribed regions [67]. They can facilitate the detection of functional variation [17] and offer the possibility of selecting markers according to the biochemical and physiological properties of the gene products in relation to the phenotype [68].

Similarly, 11,934 SNPs were detected in 4,380 of the 22,761 contigs, yielding an average of approximately 1 SNP every 500 bp. By limiting the SNP detection to the 953 contigs with high coverage (> 100×) (available in Additional file 6 in *fasta *format), 839 SNPs were identified (about 1 SNP every 1,000 bp). As expected, transitions occurred at a higher rate than transversions in both analyses, at approximately a 3:1 ratio (Table 2). Previously, ESTs (generated by Sanger sequencing) were used to describe the first set of SNPs (20 loci) for *H. midae *[10] and 11 SNPs were developed from SSR flanking regions [12]. Some of those SNPs were successfully genotyped for a population genetic analysis of wild populations of *H. midae *[63]. A selected subset of 11 SNPs detected in the contigs has also been tested for genotyping in a GoldenGate Veracode genotyping assay (Illumina) with positive results.

**Table 2:** Summary statistics for the SNPs detected in the *H. midae *library.

SNP type	Allele variations	First analysis Count/Percentage	Second analysis Count/Percentage
Transition	A↔G	3319/28.2%	262/31.2%
	C↔T	3412/29.0%	267/31.8%

Transversion	A↔C	1312/11.1%	71/8.5%
	A↔T	1777/15.1%	115/13.7%
	G↔C	741/6.3%	47/5.6%
	G↔T	1216/10.3%	77/9.2%

For each type of SNP, the number and percentage for the two analyses carried out with different parameters (see Material and Methods section) are reported.

Slightly different, but comparable levels of polymorphism were observed in the transcriptomes of other non-model animals where high throughput sequencing was used, for example in the Antarctic bivalve *Laternula elliptica *(1 SNP every 294 bp: [32]) and in the flesh fly *Sarcophaga crassipalpis *(1 SNP every 1,383 bp: [31]). The polymorphism detected in the current study (1 SNP every 500 bp) showed an intermediate level when compared to the highest level observed in *L. elliptica *and the lowest level observed in *S. crassipalpis*. In the former, the library was constructed using a similar number of animals (24) as in the current study, but collected in the wild. In the latter, the library was prepared from a long-standing laboratory colony. The fact that the abalone library originated from 19 siblings from a specific family could explain the observed trend and lead to the supposition that the SNP variation detected is likely a small fraction of that existing in natural populations of *H. midae*.

## Conclusions

The present study describes the first comprehensive transcriptomic sequence characterization of the abalone *Haliotis midae*, the most economically important aquaculture species in South Africa. More than 20,000 putative transcripts were obtained, with a large percentage matching to known proteins. This demonstrates the feasibility of the Illumina technology for *de novo *sequence assembly in a non-model species. According to the various functional categories in public annotation databases, the sample seems to be well represented and allows the identification of several genes associated with disease resistance, one of the most relevant traits for aquaculture species. Screening of the transcriptome allowed the detection of thousands of SNPs and SSRs that will be useful for future genomic studies. Finally, the high coverage showed by most of the transcripts will allow the investigation of differential gene expression with the aim of targeting genes associated with various genetic based traits. A project based on the estimation of differential gene expression patterns in relation to growth is currently being conducted in our laboratory.

## Methods

### Sampling

A total of 19 animals designated for RNA extraction and downstream transcriptome sequencing were collected from the Roman Bay Sea Farm (Gansbaai, South Africa). All animals were two-year old siblings from a specific family with a range of shell sizes between 26 and 64 mm in length. The animals were transported in oxygenated seawater to the laboratory where they were kept for no longer than 3 hours before being sacrificed. For tissue collection, animals were taken from the water one by one and placed on ice for ten minutes, shell-side down, to allow muscle contraction to slow down. This was necessary to assist fast and effective dissection. For each abalone, all mucus and water was wiped away with tissue paper and subsequently all soft tissue was dissected away from the shell and placed in a 90 mm petri dish. The tissue was cut into 5 mm strips and transferred to a tube containing RNALater solution (Ambion). For RNA isolation and cDNA library preparation, the animals were divided in three groups. The first, constituted by three specimens, was originally used for testing the next generation sequencing protocols and building the first *H. midae *reference transcriptome. The second and the third group, both constituted by eight specimens, were separated according to observed differential growth for a parallel project aimed at the investigation of differential gene expression. The sequencing output of the three groups was used to build a more comprehensive reference transcriptome.

### RNA isolation and cDNA library preparation

All glassware used during RNA extraction procedures was baked at 145°C for 6-7 hours and plastic ware were soaked in a solution of 0.1% SDS and 0.1 M NaOH at 37ËšC overnight. A protocol for extraction of cytosolic RNA adapted from [69] and [70] was followed. To convert the total RNA into a library of template molecules suitable for high throughput DNA sequencing, poly-A containing mRNA molecules were isolated, fragmented and copied into cDNA. Briefly, 11 μg (for each group described above) of total RNA was incubated at 65°C for five minutes, during which RNA secondary structures were disrupted, and mRNA was isolated using oligo (dT) magnetic beads. The 9 μl of mRNA obtained after oligo (dT) purification was fragmented by adding 1 μl of 10 × Fragmentation Buffer (Ambion) and incubated at 70°C for 5 minutes. The cleaved RNA fragments were then copied into first strand cDNA using SuperScript II reverse transcriptase (200 U/μL, Invitrogen) and a high concentration of random hexamer primers. This was followed by second strand cDNA synthesis using DNA Polymerase I and RNaseH. DNA was purified using QIAquick PCR spin columns (Qiagen) and eluted in 30 μL of Elution Buffer solution. The following part of the library preparation was performed using the Illumina Genomic DNA Sample Prep Kit according to manufacturer's instructions. In this phase, the cDNA fragments were subjected to an end-repair process followed by the ligation of the adapters. Finally, products were enriched with PCR to create the final cDNA library using two primers that anneal to the ends of the adapters. Only 18 cycles of PCR were employed, to avoid any skewing of the representation of the library (Illumina, Inc.) The amplified product was loaded onto a 2% agarose gel to verify that the correct sized template (± 300 bp) amplified. Sequencing of clustered template DNA on the Genome Analyzer was performed using four-color DNA Sequencing-By-Synthesis (SBS) technology.

### Assembly

A paired end (40 and 45 bp) and two single end raw short reads sets (45 bp) sequenced in three different lanes of the Illumina Genome Analyzer II sequencer were cleaned by removing adapter sequences, empty reads and low quality sequences (where the percentage of non-determined bases, identified by "N", was ≥ 10% of the total length). Furthermore, to avoid vector contamination, the reads with significant matches against the UniVec database http://www.ncbi.nlm.nih.gov/VecScreen/UniVec.html were deleted. Files containing the sequences and quality scores have been submitted to the National Center for Biotechnology Information (NCBI) Short Read Archive (accession number SRA024566). High quality reads of each of the three datasets (single and paired ends), were *de novo *assembled by using the CLC Genomics Workbench v4.0 software (CLCbio, Aarhus, Denmark). The program implements an algorithm that works using de Bruijn graphs by making a table of all sub-sequences of a certain length (called K-mers) found in the reads and subsequently concatenating them into longer sequences called contigs. After building contigs with all the available reads, the program uses the information stored in paired end reads to scaffold these contigs into longer sequences (see the software documentation for details). For the paired end sequences set, an insertion length ranging between 170 and 250 bp was selected as it was empirically determined after several *de novo *runs that all the assembled reads were normally distributed in this range with the highest frequency at 210 bp. Since for this work there was a requirement for longer sequences of good quality, which would enable the distinction between gene family members, only contigs with length ≥ 100 bp were considered and further analyzed. The resultant contigs were saved and their consensus sequences exported for BLAST search, annotation analysis and marker discovery.

### BLAST and Annotation

The coverage and the quality of the assembled contigs were assessed by aligning them against the Genbank non-redundant (NR) protein database using the BLASTx algorithm. BLASTn was used to align the assembled contigs against the collection of EST sequences of the NCBI (dbEST). Functional annotation in the form of gene ontology (GO) was extracted from the NR database using Blast2GO v2.4.4 [71], an automated tool for the assignment of gene ontology terms to BLAST matches (*E*-value threshold of 10^-10^). Since Blast2GO was designed to be used with novel sequence data, it was well suited to the *de novo *assembled contigs produced in this study. Furthermore, functional annotation was performed by BLAST comparisons (using the BLASTx algorithm) against the eukaryotic orthologous groups of proteins (KOG). This analysis was performed using the Desktop cDNA Annotation System (dCAS) v1.4.3 [72] using the same stringent *E*-value threshold of 10^-10^. The dCAS application was also used to annotate the sequences, by the use of the rpsBLAST algorithm, against the database of protein families and domains (PFAM) [73].

To evaluate the degree of gene conservation, dCAS was used to align the contigs against the sequence data from another mollusc species with comprehensive annotated genomic resources; the gastropod snail *Lottia gigantea *http://genome.jgi-psf.org/Lotgi1/Lotgi1.home.html. For the same purpose, the ESTs collection, the clustered ESTs (the resource more comparable with our data) and the Gene filtered model of *L. gigantea *were used as reference to map the *H. midae *short reads. Mapping was performed with the Mosaik v1.0 software (Michael Stromberg, Boston University). Different combinations of 'hash size' (15, 17 and 19) and 'number of mismatches allowed' (2, 3 and 4) were tested for each of the three *Lottia *sequence sets. The *L. gigantea *clustered ESTs were also used to evaluate the representativeness of the *H. midae *transcriptome by a comparative analysis of the annotated sequences of both mollusc species to the KOG database (where assignment of query sequences to multiple classes is limited).

All assembly, BLAST, mapping and annotation data were loaded in a local MySql database and summary statistics was extrapolated with relevant queries.

### Marker discovery

Phobos v3.3.11 [74] was used to detect tandem Simple Sequence Repeats (SSR) in the assembled contigs. The analysis was run setting the length of the repeat motifs to be searched between two and six and the minimum number of repeats equal to four. To assess the genotyping potential of the detected microsatellites, the subset of contigs containing a minimum number of SSR pattern repeats of seven, for di- and trinucleotide motifs, six, for tetra- and pentanucleotide motifs and five, for hexanucleotide motifs, was selected and primers for their amplification were designed using the web application BatchPrimer3 v1.0 [75]. To ensure a high probability of amplification, the primers designed in the SSR flanking regions had to have a GC content ranging between 40 and 70%, a melting temperature between 52 and 65°C with a maximum 2°C difference between each primers pair in which a GC clamp was imposed. The primers were positioned in order to obtain PCR products between 100 and 500 bp in length.

The CLC Genomics Workbench mapping facility was used to detect SNPs in the *Haliotis *contigs. SNPs with a minimum average quality value of surrounding bases and central base respectively of 15 and 20, a minimum coverage of 20× and a minimum variant allele frequency of 20% were enumerated. To obtain a subset with higher confidence, SNPs were filtered according to their presence in contigs characterized by a minimum of 100× average coverage using the same search parameters as for the first analysis except for the minimum coverage of the SNP position that was set at 80×.

## Ethical clearance

Studies conducted on the abalone *Haliotis midae *have been exempted from ethical clearance by Stellenbosch University Animal Care and Use Committee.

## Competing interests

The authors declare that they have no competing interests.

## Authors' contributions

PF conducted the bioinformatic analyses and drafted the manuscript. MvdM carried out the molecular biology experiments and participated in manuscript writing. RRW designed the experimental plan, provided funds and contributed to the manuscript preparation. All authors have read and approved the final manuscript.

## Supplementary Material

Additional file 1 **Contigs file**. *Fasta *file of the 22,271 sequences assembled *de novo*. Click here for file

Additional file 2 **Top BLAST matches from NCBI NR database**. BLAST results against the Genbank non-redundant (NR) protein database for all the contigs with a cut-off *E*-value of 10^-3 ^are shown. Click here for file

Additional file 3 **Transcripts related to disease resistance**. Overview of potential disease resistance-related sequences identified from *H. midae *cDNA library according to the GO, KOG and PFAM functional description. Click here for file

Additional file 4 **SSRs discovery**. *Fasta *file of the 181 sequences where 420 primer pairs were designed. Click here for file

Additional file 5 **Primer sequences for SSR loci**. Information of each SSR where primer pairs were designed. Repeat motif, number of repeats, sequences of forward and reverse primer and melting temperature are reported. Click here for file

Additional file 6 **SNPs discovery**. The information on the SNPs found in the contigs with coverage ≥100× are reported. Click here for file

## References

[B1] Shepherd SA Tegner MJ Guzmán del Próo SA Abalone of the World: Biology, Fisheries and Culture 1990 Oxford: Blackwell Scientific Publications

[B2] Slabbert R Identification of faster growth rate quantitative trait loci within abalone, *Haliotis midae*, using comparative microsatellite bulked segregant analysis PhD thesis 2010 Stellenbosch University, Genetics Department, South Africa

[B3] Britz PJ Lee B Botes L AISA 2009 Aquaculture Benchmarking Survey: Primary Production and Markets 2009 AISA report produced by Enviro-Fish Africa

[B4] Elliott NG Genetic improvement programmes in abalone: what is the future? Aquac Res 2000 31 51 59 10.1046/j.1365-2109.2000.00386.x

[B5] Hauser L Seeb JE Advances in molecular technology and their impact on fisheries genetics Fish Fish 2008 9 473 486

[B6] Massault C Bovenhuis H Haley C de Koning DJ QTL mapping designs for aquaculture Aquaculture 2008 285 23 29 10.1016/j.aquaculture.2008.06.040

[B7] Sonesson AK Meuwissen THE Testing strategies for genomic selection in aquaculture breeding programs Genet Sel Evol 2009 41 37 10.1186/1297-9686-41-37 19566932 PMC2714299

[B8] Liu ZJ Cordes JF DNA marker technologies and their applications in aquaculture genetics Aquaculture 2004 238 1 37 10.1016/j.aquaculture.2004.05.027

[B9] Hayes B Baranski M Goddard ME Robinson N Optimisation of marker assisted selection for abalone breeding programs Aquaculture 2007 265 61 69 10.1016/j.aquaculture.2007.02.016

[B10] Bester AE Roodt-Wilding R Whitaker HA Discovery and evaluation of single nucleotide polymorphisms (SNPs) for *Haliotis midae*: a targeted EST approach Anim Genet 2008 39 321 324 10.1111/j.1365-2052.2008.01728.x 18454808

[B11] Roux A Sandenbergh L Roodt-Wilding R Preliminary investigation to determine the cytotoxicity of various cryoprotectants on southern African abalone (*Haliotis midae*) embryos Cryobiology 2008 57 308 311 10.1016/j.cryobiol.2008.09.009 18845135

[B12] Rhode C Slabbert R Roodt-Wilding R Microsatellite flanking regions: a SNP mine in South African abalone (*Haliotis midae*) Anim Genet 2008 39 329 10.1111/j.1365-2052.2008.01718.x 18384463

[B13] Slabbert R Ruivo NR Van den Berg NC Lizamore DL Roodt-Wilding R Isolation and characterization of 63 microsatellite loci for the abalone, *Haliotis midae* J World Aquacult Soc 2008 39 429 435 10.1111/j.1749-7345.2008.00173.x

[B14] Slabbert R Hepple J Venter A Nel S Swart L Van den Berg NC Roodt-Wilding R Isolation and segregation of 44 microsatellite loci in the South African abalone *Haliotis midae *L Anim Genet 2010 41 332 333 10.1111/j.1365-2052.2009.02003.x 19958347

[B15] Ansorge WJ Next-generation DNA sequencing techniques New Biotechnol 2009 25 195 203 10.1016/j.nbt.2008.12.009 19429539

[B16] Andersen JR Lubberstedt T Functional markers in plants Trends Plant Sci 2003 8 554 560 10.1016/j.tplants.2003.09.010 14607101

[B17] Bouck A Vision T The molecular ecologist's guide to expressed sequence tags Mol Ecol 2007 16 907 924 10.1111/j.1365-294X.2006.03195.x 17305850

[B18] Pickrell JK Marioni JC Pai AA Degner JF Engelhardt BE Nkadori E Veyrieras JB Stephens M Gilad Y Pritchard JK Understanding mechanisms underlying human gene expression variation with RNA sequencing Nature 2010 464 768 772 10.1038/nature08872 20220758 PMC3089435

[B19] McManus CJ Coolon JD Duff MO Eipper-Mains J Graveley BR Wittkopp PJ Regulatory divergence in *Drosophila *revealed by mRNA-seq Genome Res 2010 20 816 825 10.1101/gr.102491.109 20354124 PMC2877578

[B20] Liu SP Li D Li QB Zhao P Xiang ZH Xia QY MicroRNAs of *Bombyx mori *identified by Solexa sequencing BMC Genomics 2010 11 148 10.1186/1471-2164-11-148 20199675 PMC2838851

[B21] Zenoni S Ferrarini A Giacomelli E Xumerle L Fasoli M Malerba G Bellin D Pezzotti M Delledonne M Characterization of transcriptional complexity during berry development in *Vitis vinifera *using RNA-Seq Plant Physiol 2010 152 1787 1795 10.1104/pp.109.149716 20118272 PMC2850006

[B22] Wang XW Luan JB Li JM Bao YY Zhang CX Liu SS *De novo *characterization of a whitefly transcriptome and analysis of its gene expression during development BMC Genomics 2010 11 400 10.1186/1471-2164-11-400 20573269 PMC2898760

[B23] Rosenkranz R Borodina T Lehrach H Himmelbauer H Characterizing the mouse ES cell transcriptome with Illumina sequencing Genomics 2008 92 187 194 10.1016/j.ygeno.2008.05.011 18602984

[B24] Collins LJ Biggs PJ Voelckel C Joly S An approach to transcriptome analysis of non-model organisms using short-read sequences Genome Inform 2009 21 3 14 19425143

[B25] Hegedus Z Zakrzewska A Agoston VC Ordas A Racz P Mink M Spaink HP Meijer AH Deep sequencing of the zebrafish transcriptome response to mycobacterium infection Mol Immunol 2009 46 2918 2930 10.1016/j.molimm.2009.07.002 19631987

[B26] Nowrousian M Stajich JE Chu ML Engh I Espagne E Halliday K Kamerewerd J Kempken F Knab B Kuo HC Osiewacz HD Pöggeler S Read ND Seiler S Smith KM Zickler D Kück U Freitag M *De novo *assembly of a 40 Mb eukaryotic genome from short sequence reads: *Sordaria macrospora*, a model organism for fungal morphogenesis Plos Genet 2010 6 e1000891 10.1371/journal.pgen.1000891 20386741 PMC2851567

[B27] Franchini P Slabbert R van der Merwe M Roux A Roodt-Wilding R Karyotype and genome size estimation of *Haliotis midae*: estimators to assist future studies on the evolutionary history of Haliotidae J Shellfish Res 2010 29 945 950 10.2983/035.029.0428

[B28] Pennacchio LA Rubin EM Genomic strategies to identify mammalian regulatory sequences Nat Rev Genet 2001 2 100 109 10.1038/35052548 11253049

[B29] Kim E Magen A Ast G Different levels of alternative splicing among eukaryotes Nucleic Acids Res 2007 35 125 131 10.1093/nar/gkl924 17158149 PMC1802581

[B30] Parchman TL Geist KS Grahnen JA Benkman CW Buerkle CA Transcriptome sequencing in an ecologically important tree species: assembly, annotation, and marker discovery BMC Genomics 2010 11 180 10.1186/1471-2164-11-180 20233449 PMC2851599

[B31] Hahn DA Ragland GJ Shoemaker DD Denlinger DL Gene discovery using massively parallel pyrosequencing to develop ESTs for the flesh fly *Sarcophaga crassipalpis* BMC Genomics 2009 10 234 10.1186/1471-2164-10-234 19454017 PMC2700817

[B32] Clark MS Thorne MAS Vieira FA Cardoso JCR Power DM Peck LS Insights into shell deposition in the Antarctic bivalve *Laternula elliptica*: gene discovery in the mantle transcriptome using 454 pyrosequencing BMC Genomics 2010 11 362 10.1186/1471-2164-11-362 20529341 PMC2896379

[B33] Ponder WF Lindberg DR Towards a phylogeny of gastropod molluscs: Analysis using morphological characters Zool J Linn Soc-Lond 1997 119 83 265 10.1111/j.1096-3642.1997.tb00137.x

[B34] Aktipis SW Giribet G A phylogeny of Vetigastropoda and other "archaeogastropods": re-organizing old gastropod clades Invertebr Biol 2010 129 220 240 10.1111/j.1744-7410.2010.00198.x

[B35] Haidle L Janssen JE Gharbi K Moghadam HK Ferguson MM Danzmann RG Determination of quantitative trait loci (QTL) for early maturation in rainbow trout (*Oncorhynchus mykiss*) Mar Biotechnol 2008 10 579 592 10.1007/s10126-008-9098-5 18491191 PMC2516301

[B36] Houston RD Haley CS Hamilton A Guyt DR Tinch AE Taggart JB McAndrew BJ Bishop SC Major quantitative trait loci affect resistance to infectious pancreatic necrosis in Atlantic salmon (*Salmo salar*) Genetics 2008 178 1109 1115 10.1534/genetics.107.082974 18245341 PMC2248365

[B37] De-Santis C Jerry DR Candidate growth genes in finfish - Where should we be looking? Aquaculture 2007 272 22 38 10.1016/j.aquaculture.2007.08.036

[B38] Bachere E Mialhe E Noel D Boulo V Morvan A Rodriguez J Knowledge and research prospects in marine mollusk and crustacean immunology Aquaculture 1995 132 17 32 10.1016/0044-8486(94)00389-6

[B39] Mialhe E Bachere E Boulo V Cadoret JP Strategy for research and international-cooperation in marine invertebrate pathology, immunology and genetics Aquaculture 1995 132 33 41 10.1016/0044-8486(94)00383-Y

[B40] Oakes FR Fields RC Infestation of *Haliotis rufescens *shells by a sabellid polychaete Aquaculture 1996 140 139 143 10.1016/0044-8486(95)01190-0

[B41] Li TW Ding MJ Zhang J Xiang JH Liu RY Studies on the pustule disease of abalone (*Haliotis discus hannai *Ino) on the Dalian coast J Shellfish Res 1998 17 707 711

[B42] Lizarraga-Partida ML Anguiano-Beltran C Searcy-Bernal R Vasquez-Moreno E Bacterial water quality in abalone farms of Baja California J Shellfish Res 1998 17 689 692

[B43] Nishimori E Hasegawa O Numata T Wakabayashi H *Vibrio carchariae *causes mass mortalities in Japanese abalone, *Sulculus diversicolor supratexta* Fish Pathol 1998 33 495 502

[B44] Ruck KR Cook PA Sabellid infestations in the shells of South African molluscs: Implications for abalone mariculture J Shellfish Res 1998 17 693 699

[B45] Moore JD Robbins TT Hedrick RP Friedman CS Transmission of the Rickettsiales-like prokaryote "*Candidatus xenohaliotis californiensis*" and its role in Withering syndrome of California abalone, *Haliotis *spp J Shellfish Res 2001 20 867 874

[B46] Diggles BK Nichol J Hine PM Wakefield S Cochennec-Laureau N Roberts RD Friedman CS Pathology of cultured paua *Haliotis iris *infected with a novel haplosporidian parasite, with some observations on the course of disease Dis Aquat Organ 2002 50 219 231 10.3354/dao050219 12219978

[B47] Bower SM Update on emerging abalone diseases and techniques for health assessment J Shellfish Res 2003 22 805 810

[B48] Handlinger J Carson J Donachie L Gabor L Taylor D Walker P, Lester R, Bondad-Reantaso MG Bacterial infection in Tasmanian farmed abalone: Causes, pathology, farm factors and control options Diseases in Asian aquaculture V. Proceedings of the 5th Symposium on Diseases in Asian Aquaculture: 24-28 November 2002 Australia 2002 289 300

[B49] Travers MA Le Bouffant R Friedman CS Buzin F Cougard B Huchette S Koken M Paillard C Pathogenic *Vibrio harveyi*, in contrast to non-pathogenic strains, intervenes with the p38 MAPK pathway to avoid an abalone haemocyte immune response J Cell Biochem 2009 106 152 160 10.1002/jcb.21990 19058134

[B50] The International Abalone Society (IAS) http://internationalabalonesociety.org/

[B51] Paul WE Fundamental immunology 2003 Philadelphia: Lippincott, Williams and Wilkins

[B52] Fernandes JMO Kemp GD Molle MG Smith VJ Anti-microbial properties of histone H2A from skin secretions of rainbow trout, *Oncorhynchus mykiss* Biochem J 2002 368 611 620 10.1042/BJ20020980 12164782 PMC1222992

[B53] Zasloff M Antimicrobial peptides of multicellular organisms Nature 2002 415 389 395 10.1038/415389a 11807545

[B54] Roch P Beschin A Bernard E Antiprotozoan and antiviral activities of non-cytotoxic truncated and variant analogues of mussel defensin Evid Based Complement Alternat Med 2004 1 167 174 10.1093/ecam/neh033 15480442 PMC516463

[B55] Li CH Zhao JM Song LS A review of advances in research on marine molluscan antimicrobial peptides and their potential application in aquaculture Molluscan Res 2009 29 17 26

[B56] De Zoysa M Nikapitiya C Whang I Lee JS Lee J Abhisin: A potential antimicrobial peptide derived from histone H2A of disk abalone (*Haliotis discus discus*) Fish Shellfish Immun 2009 27 639 646 10.1016/j.fsi.2009.08.007 19706329

[B57] Terahara K Takahashi KG Mechanisms and immunological roles of apoptosis in molluscs Curr Pharm Design 2008 14 131 137 10.2174/138161208783378725 18220825

[B58] Roch P Defense mechanisms and disease prevention in farmed marine invertebrates Aquaculture 1999 172 125 145 10.1016/S0044-8486(98)00439-6

[B59] Canesi L Gallo G Gavioli M Pruzzo C Bacteria-hemocyte interactions and phagocytosis in marine bivalves Micros Res Techniq 2002 57 469 476 10.1002/jemt.10100 12112429

[B60] Hepple J Linkage mapping in *Haliotis midae* MSc thesis 2010 Stellenbosch University, Genetics Department, South Africa

[B61] Bester AE Slabbert R D'Amato ME Isolation and characterization of microsatellite markers in the South African abalone (*Haliotis midae*) Molecular Ecology Notes 2004 4 618 619 10.1111/j.1471-8286.2004.00755.x

[B62] Rhode C Development of gene-linked molecular markers in South African abalone (*Haliotis midae*) using an *in silico *mining approach MSc thesis 2010 Stellenbosch University, Genetics Department, South Africa

[B63] Slabbert R Bester AE D'Amato ME Analysis of genetic diversity and parentage within a South African hatchery of the abalone *Haliotis midae *Linnaeus using microsatellite markers J Shellfish Res 2009 28 369 375 10.2983/035.028.0220

[B64] Bester-van der Merwe AE Roodt-Wilding R Volckaert FAM D'Amato ME Historical isolation and hydrodynamically constrained gene flow in declining populations of the South-African abalone, *Haliotis midae* Conserv Genet in press

[B65] Zane L Bargelloni L Patarnello T Strategies for microsatellite isolation: a review Mol Ecol 2002 11 1 16 10.1046/j.0962-1083.2001.01418.x 11903900

[B66] Hamilton MB Pincus EL Di Fiore A Fleischer RC Universal linker and ligation procedures for construction of genomic DNA libraries enriched for microsatellites Biotechniques 1999 27 500 507 10489609 10.2144/99273st03

[B67] Barbara T Palma-Silva C Paggi GM Bered F Fay MF Lexer C Cross-species transfer of nuclear microsatellite markers: potential and limitations Mol Ecol 2007 16 3759 3767 10.1111/j.1365-294X.2007.03439.x 17850543

[B68] Chee PW Rong JK Williams-Coplin D Schulze SR Paterson AH EST derived PCR-based markers for functional gene homologues in cotton Genome 2004 47 449 462 10.1139/g04-002 15190362

[B69] Carninci P Nakamura M Sato K Hayashizaki Y Brownstein MJ Cytoplasmic RNA extraction from fresh and frozen mammalian tissues Biotechniques 2002 33 306 309 12188181 10.2144/02332st01

[B70] Falcao VDR Tonon AP Oliveira MC Colepicolo P RNA Isolation method for polysaccharide rich algae: agar producing *Gracilaria tenuistipitata *(Rhodophyta) J Appl Phycol 2008 20 9 12 10.1007/s10811-007-9174-7

[B71] Conesa A Gotz S Garcia-Gomez JM Terol J Talon M Robles M Blast2GO: a universal tool for annotation, visualization and analysis in functional genomics research Bioinformatics 2005 21 3674 3676 10.1093/bioinformatics/bti610 16081474

[B72] Guo YJ Ribeiro JMC Anderson JM Bour S dCAS: a desktop application for cDNA sequence annotation Bioinformatics 2009 25 1195 1196 10.1093/bioinformatics/btp129 19318425 PMC2732306

[B73] Finn RD Mistry J Tate J Coggill P Heger A Pollington JE Gavin OL Gunasekaran P Ceric G Forslund K Holm L Sonnhammer ELL Eddy SR Bateman A The Pfam protein families database Nucleic Acids Res 2010 38 211 222 10.1093/nar/gkp985 PMC2808889 19920124

[B74] Mayer C Phobos 3.3.11 2006 http://www.rub.de/spezzoo/cm/cm_phobos.htm

[B75] You FM Huo NX Gu YQ Luo MC Ma YQ Hane D Lazo GR Dvorak J Anderson OD BatchPrimer3: A high throughput web application for PCR and sequencing primer design BMC Bioinformatics 2008 9 253 10.1186/1471-2105-9-253 18510760 PMC2438325

